# Willingness to pay for a quality-adjusted life year: an evaluation of attitudes towards risk and preferences

**DOI:** 10.1186/1472-6963-14-287

**Published:** 2014-07-03

**Authors:** Jesus Martín-Fernández, Elena Polentinos-Castro, Ma Isabel del Cura-González, Gloria Ariza-Cardiel, Victor Abraira, Ana Isabel Gil-LaCruz, Sonia García-Pérez

**Affiliations:** 1 Consultorio Local de Villamanta (C.S Navalcarnero). Gerencia de Atención Primaria. Servicio Madrileño de Salud, Red de Investigación en Servicios Sanitarios y Enfermedades Crónicas (REDISSEC), Avda de la Libertad s/n, Villamanta 28610, Madrid, Spain; 2 Unidad Docente de Atención Familiar y Comunitaria Norte. Gerencia de Atención Primaria, Servicio Madrileño de Salud. Red de Investigación en Servicios Sanitarios y Enfermedades Crónicas (REDISSEC), C/Alfonso Rodríguez Castelao, nº 17-28035 Madrid, Spain; 3 Unidad de Apoyo a la Investigación. Gerencia de Atención Primaria. Servicio Madrileño de Salud. Departamento de Medicina Preventiva y Salud Pública, Rey Juan Carlos I University. Red de Investigación en Servicios Sanitarios y Enfermedades Crónicas (REDISSEC), Avda San Martin de Porres, nº6, 28035 Madrid, Spain; 4 Unidad Docente de Atención Familiar y Comunitaria Oeste. Gerencia de Atención Primaria, Servicio Madrileño de Salud. Red de Investigación en Servicios Sanitarios y Enfermedades Crónicas (REDISSEC), C/Alonso Cano 8, 28933 Móstoles, Madrid, Spain; 5 Unidad de Bioestadística Clínica, Hospital Universitario Ramón y Cajal, IRYCIS, Servicio Madrileño de Salud, CIBER de Epidemiología y Salud Pública (CIBERESP), Ctra. Colmenar km 9.100, Madrid 28034, Madrid, Spain; 6 Departamento de Dirección y Organización de Empresas. Escuela de Ingeniería y Arquitectura, Universidad de Zaragoza, María de Luna, 3; Campus Río Ebro, 50018 Zaragoza, Spain; 7 Instituto de Salud Carlos III. Agencia de Evaluación de Tecnologías Sanitarias, Madrid, Spain, Red de Investigación en Servicios Sanitarios y Enfermedades Crónicas (REDISSEC), Av. Monforte de Lemos 5-28029 Madrid, Spain

**Keywords:** Economics, Willingness to pay, Quality-adjusted life year, Risk-taking, Contingent valuation, Primary care

## Abstract

**Background:**

This paper examines the Willingness to Pay (WTP) for a quality-adjusted life year (QALY) expressed by people who attended the healthcare system as well as the association of attitude towards risk and other personal characteristics with their response.

**Methods:**

Health-state preferences, measured by EuroQol (EQ-5D-3L), were combined with WTP for recovering a perfect health state. WTP was assessed using close-ended, iterative bidding, contingent valuation method. Data on demographic and socioeconomic characteristics, as well as usage of health services by the subjects were collected. The attitude towards risk was evaluated by collecting risky behaviors data, by the subject’s self-evaluation, and through lottery games.

**Results:**

Six hundred and sixty two subjects participated and 449 stated a utility inferior to 1. WTP/QALY ratios varied significantly when payments with personal money (mean €10,119; median €673) or through taxes (mean €28,187; median €915) were suggested. Family income, area income, higher education level, greater use of healthcare services, and the number of co-inhabitants were associated with greater WTP/QALY ratios. Age and female gender were associated with lower WTP/QALY ratios. Risk inclination was independently associated with a greater WTP/QALY when “out of pocket” payments were suggested. Clear discrepancies were demonstrated between linearity and neutrality towards risk assumptions and experimental results.

**Conclusions:**

WTP/QALY ratios vary noticeably based on demographic and socioeconomic characteristics of the subject, but also on their attitude towards risk. Knowing the expression of preferences by patients from this outcome measurement can be of interest for health service planning.

## Background

Health policies must take into consideration equity, solidarity, and accessibility, but cannot be designed without an outlook of efficiency [1]. In health economic evaluations, efficiency is usually measured using common decision rules, such as an intervention being “good value for the money” if the price of each outcome falls below a relevant threshold. This threshold represents the value of a health gain within a specific decision-making context [2].

The quality-adjusted life year (QALY) is a measure of clinical effectiveness, based on utility, which is used to compare different programs and technologies [3]. The different proposals to estimate a threshold of acceptability for a QALY are based on one of the fundamental elements of the market: supply and demand [4]. When considering supply, we can attempt to estimate the optimal price of a QALY by searching to identify the threshold of the incremental cost per QALY that the budget characteristics of the institution facing the expense could afford [5,6]. From the side of demand, considering the relevant threshold as the monetary value society places on marginal health gains, the value of the QALY can be empirically estimated with some preference elicitation method, such as the contingent valuation (CV) method. This method consists of directly asking subjects about their Willingness to Pay (WTP) for a good or service, building a hypothetical scenario where the interviewees play the role of demand and the interviewer plays the role of the supply [7]. CV has been widely used for the purpose of obtaining the values attributed to a QALY by citizens [8-14]. CV allows direct assessment of the preferences of the interviewees, which is considered valid if it is in accordance with classic economic theory [2]. However, the valuation of a QALY through CV has limitations. The construct of a QALY assumes the equality of value for all subjects regardless of their individual characteristics (income level, education, health condition, etc.). Besides, it would be expected that the metrics to measure value and health are linearly related [15]. However, the majority of published studies find that these two assumptions do not come true. The function relating WTP with potential health gains is not linear but concave [9,16] so that, even though the increase of both variables is related, they are not proportional, especially for large increases in outcome when the budget constraint appears. Additional aspects remain unclear, such as discrepancies resulting from the use of different analytical approaches [17], from evaluating different magnitudes in health condition changes, and from different question formulation [11] or periods of payment [18]. Subjects’ health condition at the study’s onset can also influence the expression of the WTP for health outcome [8]. When evaluating the results of this type of studies, the adopted perspective (ex-ante, previous to the existence of the need, versus ex-post, when the health need already exists) should also be considered.

However, there are other concerns regarding WTP/QALY expression that have not been thoroughly studied. The CV methodology is supposed to be completely deterministic regarding the health impact of the intervention as well as the relevant result. Yet, the process involves making decisions in an imaginary context that implies managing uncertainty to a degree. The respondents are asked to compare certain utility that they will obtain with the marginal utility of a quantity of money that they own at the moment. It has been shown that self reported risk-perception was related to WTP/QALY [18] but the role of attitude towards risk in WTP/QALY expression is not well known. When risk or uncertainty are introduced in the context of a good or service valuation, the results change noticeably [19,20]. Individual decision-making could be considered a function of the properties of offered choices, modulated by the subjective evaluation of the degree of risk of the tradeoff. Along these lines, a decrease in risk seeking behavior has been reported as the subject has to pay higher prices [21], and patients in need of a specific treatment that restores full health expressed a lower willingness to pay than the ex-ante estimated WTP, when individuals were risk averse [22]. For these reasons it is relevant to analyze the role of attitude towards risk in elicitation of WTP/QALY ratios.

Therefore, we find it necessary to extend the study of the threshold of acceptability for paying for a QALY: firstly, by further studying different geographical and social contexts, and secondly, by including new elements, which allow for the identification of personal characteristics that can contribute to variability in the expressed valuation. The objective of the current study is to evaluate the expression of WTP for a gained QALY by people who attend the healthcare system and assess the personal characteristics that are related to this response, paying particular attention to the attitude towards risk of the subjects. Finally, we will discuss the difficulties that may exist in establishing the limits of social acceptability through individual responses.

## Methods

### Design

Multi-centre cross-sectional study, using contingent valuation methodology.

### Studied population

The questionnaire (Additional file 1) was presented to patients 18 years of age and older, who had attended consultation in 23 health centers distributed all over the Community of Madrid (Spain), and who gave written consent within the context of a study of economic valuation of health services.

### Sample selection

The health centers were chosen for convenience, so that urban and rural environments and the upper and lower tercile of the area’s income distribution were represented. In each center, subject selection was made by random systematic sampling of appointment records, obtaining 90% of the subjects from consultation appointments at the center and 10% from home visits. Patients who did not understand the language properly and those who were not able to understand the consent form were excluded.

### Variables and measurement tools

The perception of personal health condition by the patients was evaluated using EuroQol-5D-3L. The results of EuroQol-5D-3L were expressed in the visual analog scale (VAS) and transformed into utilities by applying the results of Spanish tariffs [23]. Other variables were collected and classified into health center characteristics, patient demographic and social characteristics, health needs, pattern of usage of the health services, and attitude towards risk.

The health center’s characteristics are defined by its rural or urban environment and by the available average area income, which is classified into upper and lower tercile.

Age, sex, and nationality of the subject were recorded, their education level classified as “low” (no high-school education) or “high” (high-school education and/or higher education), “social class”, adjusted family income in thousands of Euros, number of family members living in the home, and perception of familial support as measured by the Family Apgar test. The family income was adjusted according to the method proposed by the Organisation for Economic Co-operation and Development [24]. Subjects were also asked about having additional health insurance.

With respect to health needs and usage of health services, the existence of chronic pathologies (requiring health care for a period greater than 6 months), hospitalizations over the last year, and number of visits to a primary health physician in the last year, were studied. These variables were obtained from the computerized clinic record of the patient.

In order to study attitude towards risk, we studied the existence of risky behaviors on the one hand, such as excessive alcohol consumption, smoking habits, or consumption of other drugs recorded in the clinic record. The subject’s own perception of risk predisposition was evaluated in each subject using a scale where 1 represented the maximum aversion and 10 the maximum tendency for risk. The main variable for evaluating attitudes towards risk was measured through a lottery game, adapted from the “German Socio-Economic Panel Study (SOEP)” [25]. Each subject was asked about their behavior in a game of chance that could result in a maximum loss of €40 and a maximum gain of €200. When the subject chose to continue playing even when the expected value of the lottery game was lower in probabilistic terms than the sure value, the subject was recorded as “prone to risk”.

### Willingness to pay

The WTP for a QALY was estimated by asking the patient about the maximum quantity of money they would be willing to pay monthly for a “product” that would allow them to recover perfect health condition (state 11111 in EuroQol-5D-3L). It was assumed that there was no risk of any kind from the administration of such a product, and the effect would remain while being used. WTP was valued by a closed-ended iterative bidding system [14,26]. Each subject was asked if they would be willing to pay a certain amount of their personal money per month. The bidding was randomly started from one of the two extreme values of €1/month or €8,192/month: in the first case, if subjects answered positively, then the amount was doubled until they expressed an unwillingness to pay the specified amount, and in the second case, if subjects answered negatively, then the amount was halved until they expressed a willingness to pay the specified amount. This process was later repeated but considering that the payment was to be made through new taxes rather than the subject’s personal money.

All variables were either collected from the clinic record or from the personal interview, which was carried out by a person specifically trained for it.

### Data analysis

The WTP for a QALY was calculated using the following expression:

$$\mathrm{WTP}/\mathrm{QALY}=\frac{\mathrm{WTP}/\mathrm{month}\times 12}{1-\mathrm{HRQL}}$$

where HRQL is the expression of the perception of quality of life at present, transformed into utilities. Utility values below 0 were adjusted to 0. The rest of the analyses were conducted using this dependent variable, but the WTP/QALY was also estimated using the VAS as an expression of quality of life. Those subjects stating to be in a perfect health condition were excluded.

The discount rate and subjects’ life expectancy were not taken into account when asking about the monthly WTP. First, the relationship between WTP to recover a perfect health condition and perceived health condition was evaluated, since these conditions have been proposed to be necessary for estimating the validity of the model [2].

In order to evaluate personal variability of the expressed WTP/QALY, explanative models were built where the dependent variable was the natural logarithm of WTP/QALY (lnWTP/QALY). The transformation was chosen because of asymmetry in the distribution of the raw variable.

Multilevel models were chosen to allow the study of aggregated data [27]. Each model consists of two levels, the subject and the group they belong to (the health center), and in a general way can be expressed as follows:

$$Y_{\mathrm{ij}}=\gamma_{0}+\gamma_{1}X_{\mathrm{ij}}+\beta_{2}Z_{j}+\mu_{0j}+\mu_{1j}X_{\mathrm{ij}}+\epsilon_{\mathrm{ij}}$$

Where Y_ij_ is lnWTP/QALY, X_ij_ represents the variables of each subject “i” from group “j”, Z_j_ is the set of variables of each group “j”, μ_0j_ is the random effect of the group in the measurements, μ_1j_ is the random effect of the slope of each group, and ϵ_ij_ is the random error for subject “i” from group “j”.

Initially, all the mentioned variables related to the subject’s demographic and social characteristics, health needs, and health services usage pattern, as well as economic characteristics of the area and its urban or rural environment were included, in addition to characteristics of attitudes towards risk. Those variables that did not reach significance were omitted from the model, leaving only those that maximized its explanative capability in the final model, according to the principle of parsimony. The direction of each association was crosschecked against what was expected in the proposed theoretical framework. The main independent variable was the classification of the subject as “prone to risk” in the game of lotteries.

In order to evaluate the possible distortions of linearity between WTP and gain in a QALY, the previous analyses were repeated in two subgroups, defined by people with a quality of life above or below the median in the utilities distribution.

### Ethical and legal aspects

Included patients were asked for written consent to participate in the study. All the information was processed and subsequently stored in an anonymous way, accomplishing the requirements established in national legislation. All the research process will be governed by the ethical principles contained in the Declaration of Helsinki (revision Seoul 2008). Ethical approval was obtained from the Ethics Review Board of the Hospital Universitario Fundación Alcorcón, Madrid, Spain.

## Results

Seven hundred and fifty-seven patients were offered to participate, out of which 662 accepted. The 95 patients (12.6%) who declined to participate in the study were similar in sex and age to the ones interviewed, whose characteristics are shown in Table 1.

**Table 1:** Characteristics of included patients

	**Mean (95% CI)**	**Median (IQ range)**	**Percentages (95% CI)**
Age (years)	65.4 (64.1–66.6)	69 (55–78)	
Sex (Female)			60.7% (56.9–64.5%)
Spanish nationality			95.2% (93.5–96.9%)
Superior education			37.2% (33.5–40.9%)
Social group			
Manager, Director			9.1% (6.8–11.3%)
Intermediate positions			13.3% (10.6–16.0%)
Skilled non-manual worker			26.3% (22.9–29.7%)
Skilled manual worker			23.0% (19.7–26.2%)
Partially skilled worker			11.3% (8.8–13.8%)
Unskilled manual worker			17.1% (14.1–20.0%)
Family members	2.6 (2.5–2.7)	2 (2–3)	
Adjusted family income (€1,000)	0.873 (0.833–0.912)	0.707 (0.600–1.000)	
Another insurance			16.3% (13.4–19.2%)
Chronic condition			82.9% (79.9–85.9%)
Hospitalizations			29.2% (25.7–32.8%)
Doctor consultations/year	11.5 (10.7–12.4)	9 (4–15)	
Current tobacco consumption			16.3% (13.4–19.1%)
Excessive alcohol consumption			3.8% (2.2–5.3%)
Other drug consumption			0.8% (0.1–1.4%)
Self-perceived inclination to risk (1–10)	5.0 (4.8–5.2)	5 (3–7)	
Inclination to risk			8.9% (6.7–11.2%)
VAS – EuroQol-5D-3 L	65.6 (63.9–67.4)	70 (50–80)	
EuroQol-5D-3 L Utilities	0.68 (0.66–0.71)	0.76 (0.48–1.00)	

95% CI: Confidence Interval 95%.

IQ range: Interquartile range (25–75 percentile).

VAS-EuroQol-5D-3 L: Visual Analog Scale of EuroQol-5D-3 L questionnaire.

Of the 662 subjects included (27.6%; CI 95%: 24.1–36.1%), 46 of them stated a score of 100 in the VAS (6.9%; CI 95%: 4.9–9.0%), and 119 indicated a score ≥ 90 (18.0%; CI 95%: 15.0–21.0%). The characteristics of the 479 subjects who stated not being in perfect health (measured in utilities), for whom WTP/QALY was calculated and who were included in the rest of analyses, are shown in Table 2. Usually, the interviewed patients had a self-perception of being risk-neutral and behaved as risk averse at the time of playing the lottery games.

**Table 2:** Characteristics of patients expressing a “utility” value less than one

	**Mean (95% CI)**	**Median (IQ range)**	**Percentages (95% CI)**
Age (years)	67.9 (66.5–69.3)	71 (59–80)	
Sex (female)			69.3% (65.1–73.5%)
Spanish nationality			96.0% (94.2–97.9%)
Superior education			30.9% (26.7–35.1%)
Social group			
Manager, Director			7.9% (5.4–10.5%)
Intermediate positions			12.1% (9.1–15.1%)
Skilled non-manual worker			23.4% (19.5–27.3%)
Skilled manual worker			24.0% (20.1–27.9%)
Partially skilled manual worker			13.2% (10.0–16.3%)
Unskilled manual worker			19.4% (15.8–23.0%)
Family members	2.6 (2.5–2.7)	2 (2–3)	
Adjusted family income (€1,000)	0.816 (0.774–0.859)	0.600 (0.500–1.000)	
Another insurance			14.2% (11.0–17.4%)
Chronic condition			86.1% (82.9–89.4%)
Hospitalizations			34.7% (30.3–39.0%)
Doctor consultations/year	13.0 (12.0–14.1)	10 (5–17)	
Current tobacco consumption			16.1% (12.7–19.5%)
Excessive alcohol consumption			4.2% (2.3–6.1%)
Other drug consumption			0.8% (0.2–2.1%)
Self-perceived inclination to risk (1–10)	4.8 (4.6–5.1)	5 (2–7)	
Inclination to risk			7.6% (5.1–10.1%)
VAS – EuroQol-5D-3 L	60.3 (58.3–62.3)	60 (50–80)	
EuroQol-5D-3 L Utilities	0.56 (0.54–0.59)	0.65 (0.37–0.79)	

95% CI: Confidence Interval 95%.

IQ range: Interquartile range (25–75 percentile).

VAS-EuroQol-5D-3 L: Visual Analog Scale of EuroQol-5D-3 L questionnaire.

Values of the expressed WTP/QALY are shown in Table 3. The distribution is right skewed, the mean being higher than the 75^th^ percentile in all cases. The mean WTP/QALY was over €10,000 when dealing with out of pocket payments and about €30,000 when questioning involved payment with tax money. There was no significant difference when estimating WTP/QALY ratios using the VAS or the utilities as a measure of quality of life. The WTP/QALY of those subjects with a self-perception of quality of life below the median was noticeably lower than for the rest of the subjects. The values of lnWTP/QALY using out of pocket payments fit to a normal distribution, while there was a slight asymmetry in the case of payment by taxes (0.022 by Kolmogorov-Smirnov test).

**Table 3:** Estimated values of Willingness to Pay (WTP) in Euros, per a Quality-adjusted life year (QALY)

	**Mean (95% CI)**	**10**^**th **^**Percentile**	**25**^**th **^**Percentile**	**50**^**th **^**Percentile**	**75**^**th **^**Percentile**	**90**^**th **^**Percentile**
WTP/QALY^a^ in €, out of pocket payment	10,119 (5,989–14,249)	35	132	673	3,661	14,643
WTP/QALY^a^ in €, through taxes	28,187 (19,933–6,441)	49	160	915	5,129	36,118
WTP/QALY^b^ in €, out of pocket payment	10,305 (6,317–14,293)	40	160	960	3,840	15,360
WTP/QALY^b^ in €, through taxes	28,093 (19,035–37,150)	53	192	1,280	7,680	33,353
WTP/QALY^a,c^ in €, out of pocket payment	7,626 (3,876–11,377)	26	77	366	2,214	9,019
WTP/QALY^a,c^ in €, through taxes	14,926 (9,207–20,647)	31	100	473	2,885	30,281
WTP/QALY^a,d^ in €, out of pocket payment	12,634 (5,231–20,037)	89	271	1,282	5,178	14,642
WTP/QALY^a,d^ in €, through taxes	41,559 (26,142–56,976)	114	341	1,471	8,942	82,057

^a^QALY calculated as the difference between calculated utility and perfect health.

^b^QALY calculated as the difference between the value expressed in the Visual Analog Scale (VAS) and perfect health.

^c^Only those patients with utilities below the median are considered.

^d^Only those patients with utilities above the median are considered.

A strong correlation was found between health condition and WTP to recover perfect health. A model that only took into account this explanative variable was built. The greater the health gain subjects are purchasing, the higher the WTP for it. Each increased point in the VAS was associated with an increment in WTP of €5.34 (p = 0.007) and €5.11 for each 0.01 points on the scale of utilities (p = 0.001).

WTP/QALY was not equally distributed in all health centers, when we studied the model without adjusting by the explicative variables (intraclass correlation coefficient of 6.04% or 5.31%, if the payment was to be made with out of pocket payments or through taxes, respectively).

The best explanative model for the dependent variable lnWTP/QALY is shown in Table 4. In model 1, the expressed WTP is based on out of pocket payments, and in model 2 through tax payment.

**Table 4:** Explanative models for WTP/QALY (estimated by the utilities function)

		**Model 1. Dependent variable: lnWTP/QALY with out of pocket payments**	**Model 2. Dependent variable: lnWTP/QALY through taxes**
	**Variable**	**Coefficient (95% CI)**	**Coefficient (95% CI)**
Fixed effects	High income area	**0.507 (0.101–0.913)**	0.387 (-0.095–0.868)
	Age	**-0.021 (-0.033– -0.009)**	**-0.037 (-0.051– -0.022)**
	Female	**-0.671 (-1.030– -0.311)**	**-0.558 (-0.985– -0.132)**
	High education level	**0.680 (0.234–1.127)**	**0.758 (0.228–1.287)**
	Another insurance	**0.774 (0.247–1.300)**	**0.842 (0.219–1.464)**
	Adjusted family income (€1,000)	**0.662 (0.245–1.080)**	0.824 (0.331–1.317)
	Family members	**0.124 (0.001–0.247)**	0.108 (-0.037–0.254)
	Doctor consultations/year	**0.020 (0.005–0.034)**	0.016 (-0.001–0.033)
	Tobacco consumption	**-0.515 (-0.985– -0.045)**	-0.258(-0.815–0.300)
	Alcohol consumption	**1.081 (0.164–1.997)**	**1.380 (0.294–2.466)**
	Other drug consumption	**3.192 (1.015–5.369)**	-0.465 (-2.746–1.816)
	Inclination to risk (subjective 1–10)	**0.079 (0.015–0.143)**	*0.068 (-0.008–0.144)*
	Inclination to risk (lottery games)	**0.972 (0.352–1.591)**	*0.610 (-0.113–1.333)*
	WTP values in descending order	0.222 (-0.110–0.554)	**0.501 (0.109–0.893)**
	Constant	**5.495 (4.030–6.960)**	**6.445 (4.717–8.174)**
Random Effects	Variance of the constant	0.053 (0.005–0.537)	0.079 (0.008–0.770)
	Residual variance	**3.001 (2.624–3.434)**	**4.202 (3.673–4.807)**
	Intraclass correlation coefficient	0.017	0.018
		Observations = 447	Observations = 449
		-2LL = -883.3	-2LL = -963.0
		Chi^2^(14) = 202.92	Chi^2^(14) = 182.15
		Prob > chi^2^ = 0.0000	Prob > chi^2^ = 0.0000

Significant associations: p < 0.05 indicated in bold, p < 0.1 indicated in italic.

Model 1 explains 29.5% of the intra-center variability. In it, it can be appreciated that living in high-income areas increased the mean WTP/QALY by 66% (e^0.507^ = 1.66) (CI 95%: 11–249%). People having higher educational level showed a higher mean WTP/QALY (97%; CI 95%: 26–309%), and so did people having another medical insurance (217% higher mean WTP/QALY; CI 95%: 128–367%). For every additional €1,000 in adjusted family income, the mean WTP/QALY increased by 94% (CI 95%: 28–295%). The mean WTP/QALY increased 13% for every additional family member in the home (CI 95%: 0–28%), and 2% per every additional doctor consultation (CI 95%: 1–3%).

Being a woman was associated with a decrease in the mean WTP/QALY of 49% (CI 95%: -27 – -64%). For every increase in age of 10 years, this ratio decreased by 18% CI95% (-8- -28%).

If subjects were classified as prone to risk as a result of their lottery games, the mean WTP/QALY increased an average of 264% (CI 95%: 42–490%). Subjects who considered themselves prone to risk increased their mean WTP/QALY by 8% for every additional point increase in the proposed scale (CI 95%: 2–15%). Behaviors classified as risky were also related to a higher WTP/QALY except for the case of smoking habit, since smokers showed a mean WTP/QALY 40% lower than the rest of the population (CI 95%: -4 – -63%).

In model 2, “WTP/QALY through taxes”, shown in Table 4, variables related to area income, number of family members, health services usage rate, and subjective inclination to risk as measured by lottery games or risky behaviors (except for alcohol consumption), stopped being significant. Moreover, the order of asking questions (ascending or descending) became relevant.

Table 5 shows models 3 and 4 for people with a perceived quality of life above or below the median. The mean WTP/QALY is 251% higher for patients with a basal quality of life above the median with respect to the rest (model 4 compared to model 3), even though the confidence intervals overlap.

**Table 5:** Explanative models for WTP/QALY (estimated by the utilities function)

		**Model 3. Quality of life below the median**	**Model 4. Quality of life above the median**
	**Variable**	**Coefficient (95% CI)**	**Coefficient (95% CI)**
Fixed effects	High income area	0.299 (-0.265–0.864)	**0.709 (0.1989–1.219)**
	Age	-0.013 (-0.032–0.006)	**-0.017 (-0.033– -0.001)**
	Female	**-0.956 (-1.486– -0.426)**	-0.285 (-0.762–0.193)
	High education level	**0.801 (0.100–1.501)**	0.465 (-0.102–1.032)
	Another insurance	**1.292 (0.524–2.060)**	0.407 (-0.276–1.090)
	Adjusted family income (€1,000)	*0.659 (-0.004–1.322)*	**0.667 (0.158–1.177)**
	Family members	0.025 (-0.159–0.210)	**0.204 (0.045–0.362)**
	Doctor consultations/year	**0.026 (0.005–0.046)**	**0.031 (0.010–0.051)**
	Tobacco consumption	**-0.925 (-1.655– -0.195)**	-0.067 (-0.656–0.523)
	Alcohol consumption	**0.748(-0.877–2.374)**	**1.124 (0.068–2.181)**
	Other drug consumption	**4.654 (1.737–7.571)**	0.931 (-2.514–4.375)
	Inclination to risk (subjective 1–10)	**0.093 (0.003–0.183)**	0.063 (-0.025–0.152)
	Inclination to risk (lottery games)	0.218 (-0.916–1.353)	**1.151 (0.447–1.854)**
	WTP values in descending order	0.401 (-0.071–0.874)	-0.046 (-0.496–0.405)
	Constant	**4.646 (2.381–6.910)**	**5.566 (3.732–7.399)**
Random Effects	Variance of the constant	0.082 (0.005–1.432)	7×10^-12^ (6×10^-20^- 0.001)
	Residual variance	**2.927 (2.407–3.560)**	**2.627 (2.161–3.192)**
	Intraclass correlation coefficient	0.026	0.017
		Observations = 224	Observations = 223
		-2LL = -440.8	-2LL = -425.9
		Chi2(14) = 124.64	Chi2(14) = 87.99
		Prob > chi2 = 0.0000	Prob > chi2 = 0.0000

Significant associations: p < 0.05 indicated in bold, p < 0.1 in italic.

The WTP is estimated by payment with out of pocket payments in patients with perceived quality of life above or below the median.

When the hypothesis of variability of the slopes was tested, it was ruled out for all models, indicating that the relationship between risk inclination in the lottery games and WTP/QALY showed the same magnitude in all health centers. The random effect did not reach significance for any of the adjusted models, allowing for the rejection of the hypothesis that the mean effect of risk inclination on WTP/QALY was different in each center.

The analyses of the distribution of residuals in models 1, 3, and 4 fit to a normal distribution. In model 2 the Kolmogorov-Smirnov test showed a p = 0.037, even though the fitting was graphically acceptable.

## Discussion

The expression of the value for a QALY for the studied social group shows large variability, which is partially explained by personal characteristics, among which we highlight attitude towards risk.

When evaluating cumulative individual responses, we find values that differ significantly from the ones accepted by consensus (€30,000/QALY; $50,000/QALY) [28,29], especially when referring to out of pocket payments. The reported values for the WTP/QALY are very variable, but there are common findings to all of them. The majority of studies find mean WTP/QALY values below the “consensus threshold” when an ex-post perspective, such as ours, is used [8-10,14]. The ex-ante perspective (previous to the existence of the need, for example when evaluating an insurance) could incorporate altruistic and optional values to utility of outcomes with respect to the ex-post perspective (WTP is evaluated from an already existing health need), but its results are strongly influenced by the probability of the occurrence of the health problem [8]. The proposed scenario did not stress decreases in mortality risk, which increase the WTP/QALY significantly [9,10,29]. The expressed values are also noticeably lower compared to others stated in our country when subjects were questioned about “imagined” health conditions [11]. It is possible that the real experience of chronic health condition results in a certain “adaptation” [8,30] that decreases the utility of the proposed gain.

In terms of characteristics that relate to a higher WTP for a QALY, we find commonality with characteristics associated with higher stated WTP for health services. The wealth as an explanative factor of the WTP was foreseeable from the theoretical point of view [31,32] and is systematically shown in the literature [33,34]. Subjects with higher payment capacity show higher WTP/QALY. Those subjects living in high-income areas also express higher WTP/QALY, since the marginal utility of money is lower. It has been repeatedly proven that the WTP/QALY increases with the wealth of the subject [9,10,12,13,35], which suggests conformity of the answers with economic theory. Education level has also been related to higher WTP for both health services [33,36] and improvements in health condition [10,12]. On the other hand, women and older people usually express a lower WTP for the same good [32,33], an effect that remains when evaluating health condition improvements [8,10]. Perhaps this decrease with age is due to lower life expectancy or adaptation to health conditions imposing some limitations. The number of consultations per year, or in other words more intensive use of health services, is related to a higher expressed WTP/QALY.

The experience in direct payment for health services, as it occurs in people with a additional insurance (general public insurance plus private insurance), was related to higher WTP/QALY in the model. It is known that experience in the market eliminates certain distortions, possibly related to attitudes towards risk [37].

The impact of risk context on elicited WTP/QALY values has recently been suggested [20]. When studying attitudes towards risk and WTP/QALY we hypothesized that subjects that are prone to risk should express a higher WTP/QALY when asked about out of pocket payments, as the tradeoff between their own money and the forthcoming health improvement is associated with a certain degree of uncertainty. This idea was corroborated by our results and a higher WTP/QALY was found in people who define themselves as prone to risk, or behave in such a manner, with the exception of tobacco addiction. Risk inclination, both self-declared and shown in the lottery game experiments, was the strongest characteristic explaining the variability of the WTP/QALY when subjects were questioned about payment with their own money. This relationship was lost when questioning with payment through taxes, as it was expected. The case of tobacco addiction is a particular one, since smokers show a higher preference for the present, which characterizes people who are prone to risk. However, the relationship between attitude towards risk and the behavior of tobacco users is different when asking about their own perceptions (positive correlation) or with lottery games (negative correlation) [38]. On the other hand, smokers can be against new health expenses as a result of having seen themselves in the center of the debate on health expenditure repercussions of diseases derived from tobacco addiction.

There are differences between the results obtained when payment is suggested via out of pocket payments or taxes. On the one hand, variables related to risk inclination partly lose relevance, as tax payment could be considered as a type of insurance (all people share expenses). On the other hand, the order in which the questions about WTP/QALY were asked becomes relevant, showing higher values when the higher limit is the departure point, leading us to hypothesize that the limitation of payment capacity could partially disappear.

The differences of the WTP/QALY stated by people who can obtain greater potential improvements in their health condition relative to those with minor potential benefits could be the result of limitations from the budget constraints. This may also be explained as an expression of the “sub-additive” bias [2], or from risk-aversion behavior in the face of health gains.

### Limitations

The chosen population is not necessarily representative of the entire region, but only of those who seek health care from the national healthcare system, and the results are restricted to respondents who reported not being in perfect health.

At the time this study was conducted, healthcare coverage was universal and free at the moment of use, with approximately two thirds of the general population attending the health centers over a one-year period. The perception of the stated health condition is slightly lower than that of the general population in our environment, but higher than those with chronic pathologies [23]. All the social and cultural strata were represented.

The proposed scenario did not include a broad time horizon or life expectancy, according to the classic concept of a QALY at a specific time point [3]. This may qualify the results since health gain is appraised differently when changes in the quantity of life gained, and not only the gained quality of life, are included [35,39]. Other limitations of the study are typical of the methodology employed. It may be difficult to discard the presence of the hypothetical bias (yeah-saying bias). The differences in the answers when the cost was to be paid with the subjects’ personal money or via taxes indicate that subjects made a realistic valuation of the money they offered. The fact that the order of the question (ascending-descending) did not have an influence on out of pocket payments but did when paid through taxes, points in the same direction. Strategic biases, or the expression of lower values by subjects in order to lower the cost of the evaluated good, were unlikely to be observed because of dealing with scenarios that are not going to translate into reality in the immediate future.

Nevertheless, there are distortions of the individual expressed values that act in opposing directions. It is possible that sick patients overvalue the price of a QALY when talking about improving their own disease relative to healthy patients [40], but it is also true that, when valuing a specific health condition, those who suffer from it can undergo a certain adaptation [3,8,10,30].

This study has taken an individual perspective, asking respondents to value changes in their own health. However, a threshold of social acceptability cannot be directly obtained from adding the values of the expressed WTP/QALY. The “social value” pertains to the value of a QALY gained for some members of society and generated through a collectively funded healthcare system. Social valuations may be lower or higher than individual valuations [41]. We should also note that only the “demand” part of the scenario has been considered, and we have not addressed other aspects of the “supply” side of the problem. In addition, we have not studied the characteristics and budget constraints in our healthcare system to identify the threshold of the incremental cost per QALY that the society could afford.

The present study confirms the shortcomings of CV for obtaining a threshold value of acceptability for a WTP/QALY. It has been proven that the WTP/QALY varies with regards to certain personal characteristics, among which we highlight attitudes towards risk in addition to demographic and social ones. In the light of these facts, we must consider the difficulty of articulating two different theoretical models. CV allows the expression of personal preferences, fitting the expectations of welfare theory, while measuring health outcome in QALYs derives from an extra-welfare conception, which centers on maximizing health and not welfare, assuming expressions that do not change depending on the individual characteristics of the subject [3,15]. However, we consider that the attempts to estimate a threshold of acceptability for a QALY cannot be indifferent to the expression of the preferences of the public evaluating it, as the study of these issues seems to offer us a better understanding of the ways in which people value health improvements. In the near future, this line of investigation should be developed, trying to adapt the theoretical construct of QALYs to the observed reality.

## Conclusions

The current study shows that WTP for a QALY varies noticeably in our context according to demographic and socioeconomic characteristics of the subject, but also depending on their attitude towards risk. When both non-use values and life expectancy are not considered, the reported value for a QALY is below the usual thresholds accepted in our setting. This difference is more significant when evaluation is performed based on real health conditions. Even though some conditions of the classic model of QALY do not come true, these types of studies provide valuable contributions since patient preferences should be incorporated into decision-making and health service planning to the maximum possible extent.

## Competing interests

The authors declare that they have no competing interests.

## Authors’ contributions

JMF conceived, designed and performed the experiments, analyzed the data and wrote the first version of the manuscript. EPC designed and performed the experiments, analyzed and interpreted the data and reviewed and approved the manuscript. MICG designed and performed the experiments, analyzed and interpreted the data and reviewed and approved the manuscript. GAC designed and performed the experiments, analyzed and interpreted the data and reviewed and approved the manuscript. VA analyzed the data contributed to the interpretation and discussion of the results and reviewed and approved the manuscript. AIGL designed the experiments, contributed to the interpretation and discussion of the results and reviewed and approved the manuscript. SGP analyzed the data, contributed to the interpretation and discussion of the results and reviewed the final manuscript. All authors read and approved the final manuscript.

## Authors’ information

JMF MSc Health Economics, Ph D, MD, Family Physician. EPC. MSc Public Health MD. Family Physician. Preventive Medicine and Public Health Specialist. MICG MSc Public Health, PhD, MD, Family Physician. Associate Professor. GAC MSc in Public Health MD. Preventive Medicine and Public Health Specialist. VA PhD. Bioestatistics. AIGL PhD. Economist. Professor. SGP MSc. Health Economics. Pharmacist.

## Pre-publication history

The pre-publication history for this paper can be accessed here:

http://www.biomedcentral.com/1472-6963/14/287/prepub

## Supplementary Material

Additional file 1 Questionnaire. Click here for file
