# Supplementary material for: Willingness to pay for a quality-adjusted life year: an evaluation of attitudes towards risk and preferences
Source: BMC Health Serv Res. 2014 Jul 3;14:287. doi: 10.1186/1472-6963-14-287 (PMC4083040; doi:10.1186/1472-6963-14-287)
Supplement: Additional file 1 — Questionnaire. [file 1472-6963-14-287-S1.pdf]

This questionnaire was co-administered with other one for the study "Economic value perceived by the user of nurse consultations in primary care setting", between October 2011 and January 2012.

Section 0 (filled by the nurse)

Your age is ___**years**

Man **Woman**

In the last year, has the patient been admitted to a hospital? (including >24 hours stay in the emergency room)

 Yes **No**

Number of visits to the nurse in the last year: __times.

Number of visits to the family physician in the last year: __times.

The patient has a chronic pathology:

Yes No

Total number of chronic pathologies: ___

**Has the patient been diagnosed as a smoker?**	Yes	No
**Has the patient been diagnosed as a former smoker?**	Yes	No
**Has the patient been diagnosed as at risk-drinker?**	Yes	No
**Has the patient been diagnosed as alcohol-depending?**	Yes	No
**Has the patient stated consumption of other drugs?**	Yes	No

Section 1

We are going to ask you for information about yourself and your state of health

You are from …?

Spain

The rest of the European Union, including Iceland, Norway and Switzerland.

Latin America

North Africa

Sub-Saharan Africa

Other non-EU European countries

Includes: Turkey, Croatia, Macedonia, Albania, Armenia, Azerbaijan, Byelorussia, Bosnia, Georgia, Liechtenstein, Moldavia, Montenegro, Russia, Serbia, and Ukraine.

Asia

Other…

If you were not born in Spain, during how many years have you been living in Spain? ___ years

Do you have additional health insurance?

Yes No

Which one?

Asisa

Sanitas

Adeslas

Other (specify):

If you do have additional health insurance, who pays for it?

Your company Yourself

By placing a checkmark in one box in each group below, please indicate which statements best describe your state of health today.

Mobility:

I have no problems walking

I have some problems walking

I have to stay in bed

Personal care:

I have no problems with personal care

I have some problems to wash and dress myself

I cannot wash or dress myself

Daily activities (e. g., work, study, housework, family activities or during leisure time):

I have no problems doing my daily activities

I have some problems doing my daily activities

I cannot perform my daily activities

Pain/discomfort:

I have no pain or discomfort

I have moderate pain or discomfort

I have significant pain or discomfort

Anxiety/depression:

I am not anxious or depressed

I am moderately anxious or depressed

I am very anxious or depressed

Compared with my general state of health during the last 12 months, my state of health today is:

Better

Same

Worse

To help people describe how good or bad their state of health is we have drawn a scale similar to a thermometer on which 100 marks the best state of health imagined and 0 the worst state of health imagined. We would like you to indicate on this scale, in your opinion, how good or bad your state of health is *today*.

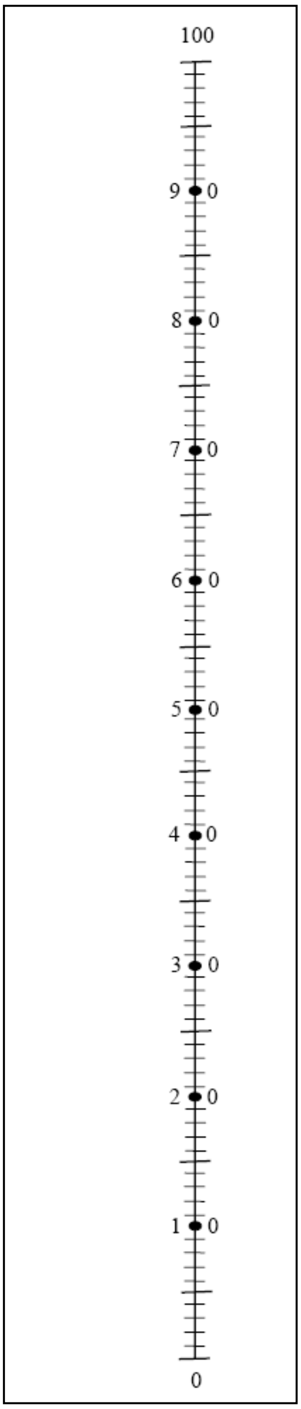

Better State of Health

Your health status today

Worst State of Health

Section 2

Next, we are going to ask you a series of questions about your acceptance or rejection of risk. There is no right or wrong answer, and we would only like to know your attitude towards certain situations.

Would you say you're risk-averse or risk-prone when there is a possible benefit? Please indicate on the scale from 1 to 10, how much of a risk-taker do you consider yourself?

Totally risk-averse = 1
Totally risk-prone = 10

1	2	3	4	5	6	7	8	9	10

Now imagine that you can take part in a lottery game in which you can choose between one of two boxes. But first, the quiz director offers you increasing amounts of money if you stop playing . One box contains €200 and the other one is empty. If you choose to compete, your prize will be the contents of the box you choose.
Mark your preferences in each situation:

□ They offer you €40 versus One of the two boxes □
□ They offer you €70 versus One of the two boxes □
□ They offer you €100 versus One of the two boxes □
□ They offer you €130 versus One of the two boxes □
□ No response

Imagine now that the lottery game rules have changed. The quiz director will also offer you increasing amounts of money each time. But, if you choose to compete you must first pay a fixed amount of €40. The prize for competing is the same as above, the contents of the box you choose, knowing that one contains €200 and the other one is empty.
Mark your preferences in each situation:

□ They offer you €0 versus One of the two boxes □
□ They offer you €30 versus One of the two boxes □
□ They offer you €60 versus One of the two boxes □
□ They offer you €90 versus One of the two boxes □
□ No response

Section 4

Finally, we are going to ask you for some statistical information that will help us to classify your answers and to interpret the results of the study:

The number of people who live in my home is: ____ persons

The number of them who are sixteen or older _____ .
The number of them who are fifteen or younger _____.

Your main occupation at present is:

Housewife.
Student.
Worker.
Unemployed.
Retired.

The highest level of education you have completed is:

I do not know how to read or write.
No education.
Primary studies.
Secondary studies.
Superior studies.

My last paying job was:

I	Manager, director, Higher professional occupations.
II	Mid-level position or sales manager.
III	Non-manual skilled worker.
IVa	Skilled manual worker.
IVb	Partially-skilled manual worker.
V	Non-skilled manual worker.

In the case of not having a paid job, the member with the highest career category within the family unit is considered. Unemployed, temporary or permanent incapacitated, or retired are classified according to the last job held.

The monthly income of your family unit (adding up all family members who live in your home) is in the range indicated with the letter:

A: Less than 600 euros

B: 600- 1200 euros.

C: 1200-1800 euros.

D: 1800-2400 euros.

E: 2400- 3600 euros.

F: 3600- 4800 euros.

G. 4800-6000 euros.

H: 6000-7200 euros.

I: More than 7200 euros.

Section 5

Finally, we are going to ask you about two imaginary scenarios

Imagine that we have a new drug that, when taken daily, allows you to return to a perfect health state (no pain, perfect mobility, total autonomy for personal care and daily activities, and absence of anxiety or depression). Imagine that the effect lasted only while you are taking it, and it has no side effects. If the new drug is not funded by the national health system and you must pay for it with your own money....

I would buy the new drug if it cost…*:

1 €/month	☐ Yes☐ No	128 €/month	☐ Yes☐ No
2 €/month	☐ Yes☐ No	256 €/month	☐ Yes☐ No
4 €/month	☐ Yes☐ No	512 €/month	☐ Yes☐ No
8 €/month	☐ Yes☐ No	1,024 €/month	☐ Yes☐ No
16 €/month	☐ Yes☐ No	2,048 €/month	☐ Yes☐ No
32 €/month	☐ Yes☐ No	4,096 €/month	☐ Yes☐ No
64 €/month	☐ Yes☐ No	8,192 €/month	☐ Yes☐ No

Now imagine that the same drug must be funded by the government through new taxes- this means that we all have to pay extra taxes to have this drug available for the entire population.

In your opinion, would it be worth funding the new drug if the cost was (for each person with the same health status as yours)... *:

1 €/month	☐ Yes☐ No	128 €/month	☐ Yes☐ No
2 €/month	☐ Yes☐ No	256 €/month	☐ Yes☐ No
4 €/month	☐ Yes☐ No	512 €/month	☐ Yes☐ No
8 €/month	☐ Yes☐ No	1,024 €/month	☐ Yes☐ No
16 €/month	☐ Yes☐ No	2,048 €/month	☐ Yes☐ No
32 €/month	☐ Yes☐ No	4,096 €/month	☐ Yes☐ No
64 €/month	☐ Yes☐ No	8,192 €/month	☐ Yes☐ No

* The interviewer begins by providing the higher or lower Euro amount depending on the random number table attached. In either case, the interviewer has to state the maximum value accepted in each situation
